# Effects of photoperiod and food on glucose intolerance and subsequent ocular pathology in the fat sand rat

**DOI:** 10.1038/s41598-023-44584-8

**Published:** 2024-01-03

**Authors:** Carmel Bilu, Neta Butensky, Amit Richter Malamud, Haim Einat, Paul Zimmet, Ofira Zloto, Hana Ziv, Noga Kronfeld-Schor, Vicktoria Vishnevskia-Dai

**Affiliations:** 1 School of Zoology, Tel-Aviv University, 69978 Ramat Aviv, Tel Aviv, Israel; 2 School of Behavioral Sciences, Tel Aviv-Yaffo Academic College, Tel-Aviv, Israel; 3 Department of Medicine, Monash University, Melbourne, VIC Australia; 4 Ocular Oncology, The Goldschleger Eye Institute, The Chaim Sheba Medical Center, Tel-Hashomer, Sackler Faculty of Medicine, Tel-Aviv University, Tel Aviv, Israel; 5 Maurice and Gabriela Goldschleger Eye Research Institute, The Chaim Sheba Medical Center, Tel-Hashomer, Sackler Faculty of Medicine, Tel-Aviv University, Tel Aviv, Israel

**Keywords:** Diabetes complications, Experimental models of disease

## Abstract

Type 2 diabetes mellitus (T2DM) and its ocular complications, such as cataract and diabetic retinopathy (DR) have been linked to circadian rhythm-disturbances. Using a unique diurnal animal model, the sand rat (*Psammomys obesus*) we examined the effect of circadian disruption by short photoperiod acclimation on the development of T2DM and related ocular pathologies. We experimented with 48 male sand rats. Variables were day length (short photoperiod, SP, vs. neutral photoperiod NP) and diet (standard rodent diet vs. low-energy diet). Blood glucose, the presence of cataract and retinal pathology were monitored. Histological slides were examined for lens opacity, retinal cell count and thickness. Animals under SP and fed standard rodent diet (SPSR) for 20 weeks had higher baseline blood glucose levels and lower glucose tolerance compared with animals kept under NP regardless of diet, and under SP with low energy diet (SPLE). Animals under SPSR had less cells in the outer nuclear layer, a lower total number of cells in the retina, and a thickened retina. Higher blood glucose levels correlated with lower number of cells in all cellular layers of the retina and thicker retina. Animals under SPSR had higher occurrence of cataract, and a higher degree of cataract, which correlated with higher blood glucose levels. Sand rats kept under SPSR develop cataract and retinal abnormalities indicative of DR, whereas sand rats kept under NP regardless of diet, or under SPLE, do not. These ocular abnormalities significantly correlate with hyperglycemia.

## Introduction

One threatening aspect of type 2 diabetes mellitus (T2DM) is the development of visual impairment^1^. Diabetes can affect all ocular structures, with cataract being the most common ocular complication and it constitutes the leading cause of blindness worldwide^2^. Diabetic retinopathy (DR) is another complication of T2DM and is the most common cause of blindness in people over the age of 50^3,4^. Better understanding of these common pathologies and the development of interventions demands research using appropriate animal models.

Most animal studies on T2DM and its ocular complications, use nocturnal mice and rats as animal models^3^. Circadian rhythm dysfunction at structural, physiological, metabolic and cellular levels, plays a critical role in the development of T2DM, diabetic cataract and DR, as a part of a wider syndrome named “The circadian syndrome”^5–7^. Considering that the circadian systems of nocturnal and diurnal mammals differ significantly, it has been repeatedly argued that diurnal model animals are more suitable for the study of circadian rhythm-related diseases, such as T2DM and its complications^5,7^. Moreover, the ocular structure and function differ significantly between diurnal and nocturnal species. Nocturnal mammals, have much larger numbers of rods within the outer nuclear layer (ONL), than diurnal species, to maximize photon catch^8^. Nocturnal species have low proportions of cone photoreceptors, which would be less functionally important in low light levels. Both mice and rats have very rod-dominated retinas with only few cones^9,10^), whereas diurnal species, like sand rats (*Psammomys obesus*), display a relatively high cone percentage in their retinas^11^. Humans have a low cone percentage, which are concentrated within the central macula affording high acuity vision under wide photopic conditions^8^, whereas in nocturnal mammals these cones are sparsely distributed across the retinal surface and not concentrated is a central macula-like structure^11,12^.

In recent studies, we found that keeping diurnal sand rats in the laboratory, under conditions mimicking human's modern lifestyle, leads to dampened behavioral and biological rhythms. This circadian desynchrony can trigger T2DM, as well as obesity, cardiometabolic diseases (CMD), depression, anxiety-like behaviors and cataract^7^. The development of these disorders is accelerated under short photoperiod conditions (SP) and standard rodent diet (SRD) (which has higher caloric density than the sand rat's natural diet)^7,13–17^.

Due to the emerging evidence supporting the importance of circadian desynchrony in the pathogenesis of the circadian syndrome, including T2DM and visual impairment, the current study explores the effects of this interaction on the retina and lens of sand rats. We hypothesized that as in previous studies^7^, sand rats kept under SP will develop circadian disruption which, when complimented with SRD (SPSR) will result with hyperglycemia and glucose intolerance. We hypothesized that if ocular pathologies such as cataracts and DR result only from the diabetic conditions, they will be evident only in the SPSR group. If they result from the circadian disruption alone, they will appear in the two SP groups regardless of diet, and if from combined effects, the ocular pathologies will be evident in the SP with low energy diet group (SPLE), and will be more severe in the SPSR group.

## Materials and methods

### Animals

Forty-eight male sand rats, 6 months old, from Tel-Aviv University Zoological garden colony, were used, individually housed in plastic cages (42cmx26cmx40cm) in temperature-controlled rooms (25°C). Before the onset of experiments, animals were maintained on a low-energy diet (LED) (product 1078, Koffolk Ltd, Israel). All animals were weighed and tested for oral glucose tolerance (OGTT) before the experiment. Animals were assigned to experimental groups based on weight and blood glucose levels to avoid baseline bias. Body weight was measured weekly.

**Figure 1:**
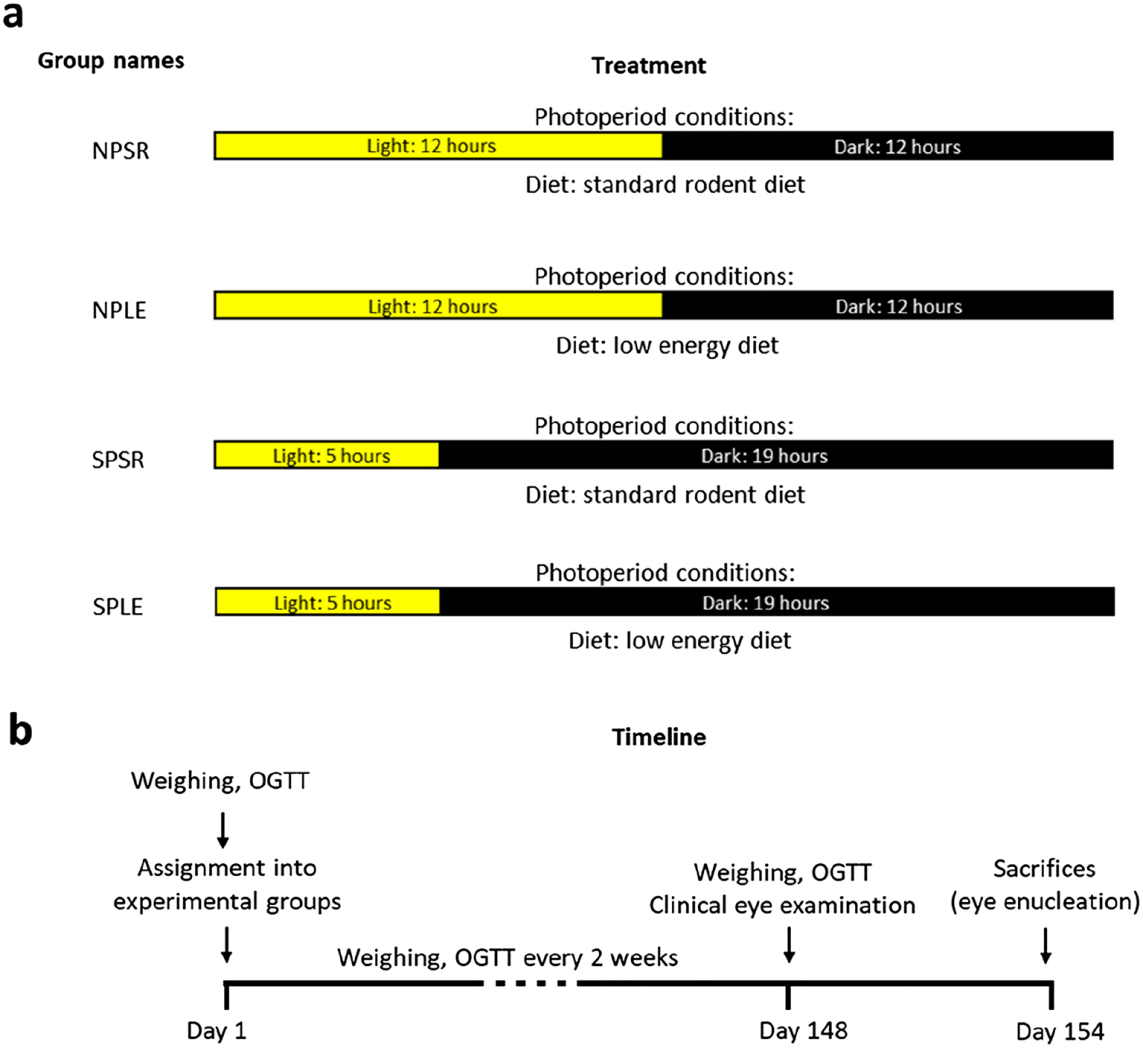
Experimental design (**a**) and timeline (**b**).

Every 2 weeks, the animals were fasted for 4 h during the light period and blood samples were collected to measure baseline circulating glucose (U-Right glucometer TD-4269, TaiDoc, Taiwan). OGTT were performed at zeitgeber time 2 (ZT2, ZT0: time of lights-on) as described in previous work^7^ (Fig. 1a,b). All experimental procedures followed the NIH guidelines and were approved by the Institutional Animal Care and Use Committee (IACUC) of Tel-Aviv University (permit L15055).

### Experimental procedure

The experiment was structured in a 2 × 2 design, with day length (SP vs. neutral photoperiod, NP) and diet (SRD vs. LED) as independent main variables (n = 12 per group). OGTT were performed on week 20. Clinical eye examination was conducted on week 21, at ZT 2. On week 22, at ZT 2, the sand rats were anesthetized, their eyes were enucleated, and processed.

### Photoperiod conditions

Sand rats were acclimated to SP (5 h light:19 h dark) NP (12 h light:12 h dark). Light illuminance was 800 lx at wavelength 420–780nm (5839K). The photoperiod regimen was chosen based on previous results demonstrating that SP results in the development of the Circadian Syndrome including depression- and anxiety-like behaviors, acceleration of the development of T2DM and CMD compared with NP^7,18^. This regimen was also based on the activity pattern of sand rats in nature, where they are active for 5h around midday during winter^19,20^.

### Clinical eye examination

Local anesthetics was achieved with administration of Oxybuprocaine hydrochloride 0.4% (Localin, Fisher Pharmaceuticals Labs, Israel).

The pupils were dilated using topical administration of one drop of 0.5% tropicamide (Mydramide, Fisher Pharmaceuticals Labs, Israel) and one drop of Phenylepheine hydrochlorie 10% (Erin 10% 5 ml Fisher Pharmaceuticals Labs, Israel).

Slit lamp examination (Keeler PSL classic portable lamp) was performed to evaluate lens opacity^21^.

Indirect ophthalmoscopy fundus examination (Welch-Allyn 12,500 Binocular Indirect Ophthalmoscope) with 20D lens (Volk Optical Inc), was performed to evaluate the retina and optic nerve.

The ophthalmic examination was performed in a masked fashion (ophthalmologist was unaware of the treatment methods).

**Figure 2:**
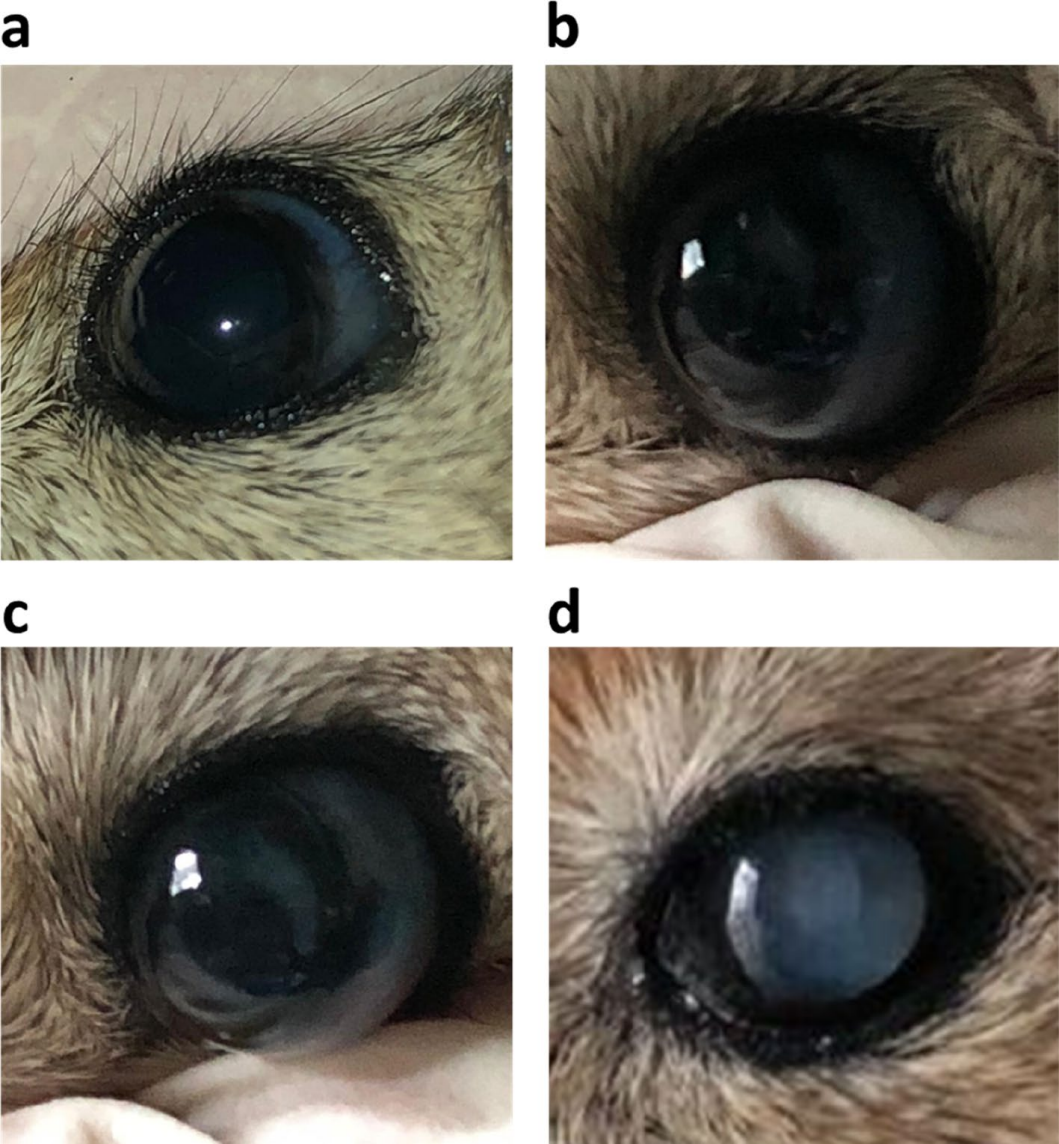
Cataract grading: Grade 0—a clear lens (**a**). Grade 1—incipient cataract, minimal opacification of the lens (**b**). Grade 2—opacification of the nucleus or cortical lens, partially obscuring the retina (**c**). Grade 3—mature cataract, dense opacification of the lens, obscuring the view of the retina.

Cataract was defined as lens opacity (ICD-9 code 366.9): A clear lens was graded as 0 (Fig. 2a). Incipient cataract was defined as minimal opacification of the lens, and graded as 1 (Fig. 2b). Cataract was defined as opacification of the nucleus or cortical lens partially obscuring the retina and graded as 2 (Fig. 2c). Mature cataract was defined as dense opacification of the lens obscuring the view of the retina and graded as 3 (Fig. 2d).

### Histopathological examination

Sand rats were sacrificed on week 22. Eyes were the enucleated and immersed in 4% paraformaldehyde for fourty-eight hours and infiltrated with a solution of 2% PFA and 5% sucrose for 1h. Then, eyes were incubated in sucrose solutions (5%, 10%, 12.5%, and 15% sucrose) for thirty minutes and then incubated in 20% sucrose for sixteen hours. Eyes were embedded in 20% sucrose in optimum cutting temperature (O.C.T, Sakura Finetek Inc., USA)^22^. Ten-micrometer cryosections along the vertical meridian of the eye through the optic nerve were cut on a cryostat followed by sagittal cuts of the two halves. As the sand rat has no macula and the cones are spread throughout all the retina, from each axial histological slide, 3 random different areas were sampled. From each sagittal histological slide, 2 random different areas were sampled. The sections were stained with hematoxylin–eosin.

**Figure 3:**
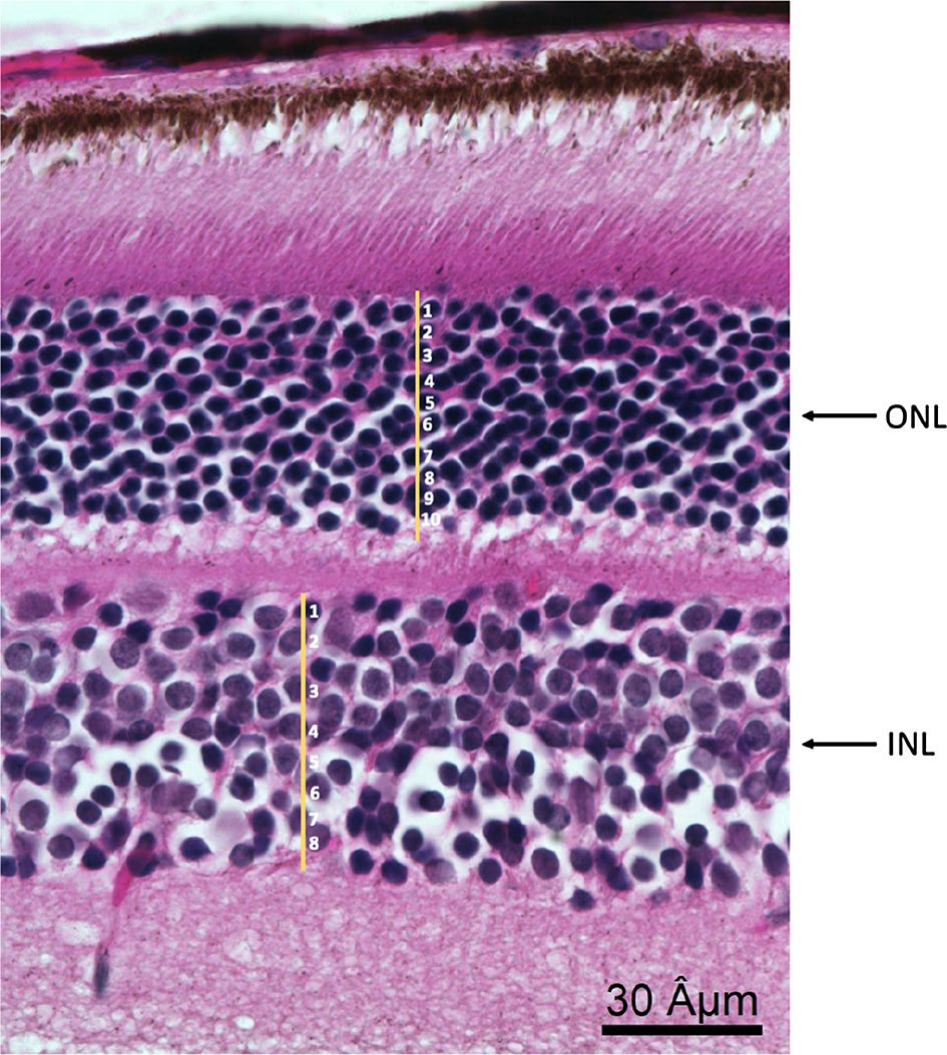
Cell count in the INL and ONL.

Hematoxylin and Eosin slides were examined using an Olympus BX60 light microscope (Olympus Optical Co Ltd, Japan). Pictures were taken using a Qimaging Retiga CCD Color Camera (Teledyne Photometrics, AZ). We counted the cells in the inner nuclear layer (INL) and outer nuclear layer (ONL) of the retina using a 60X magnification, by randomly drawing a vertical line within each layer and counting the number of cell nuclei, which touched the line (Fig. 3).

The thickness of the retina, including the outer and inner nuclear layers, outer and inner plexiform layers, and the ganglion cell layer were measured in μm, under a 20X magnification, using Stereo Investigator (MBF Bioscience, USA).

### Data analysis

Statistical analysis was performed using Statistica 13.2 (TIBCO Software, CA). Factorial ANOVA was used to analyze data from all experiments followed by LSD post-hoc tests.

### Ethical approval

The study is reported in accordance with ARRIVE guidelines.

## Results

### Baseline blood glucose levels

**Figure 4:**
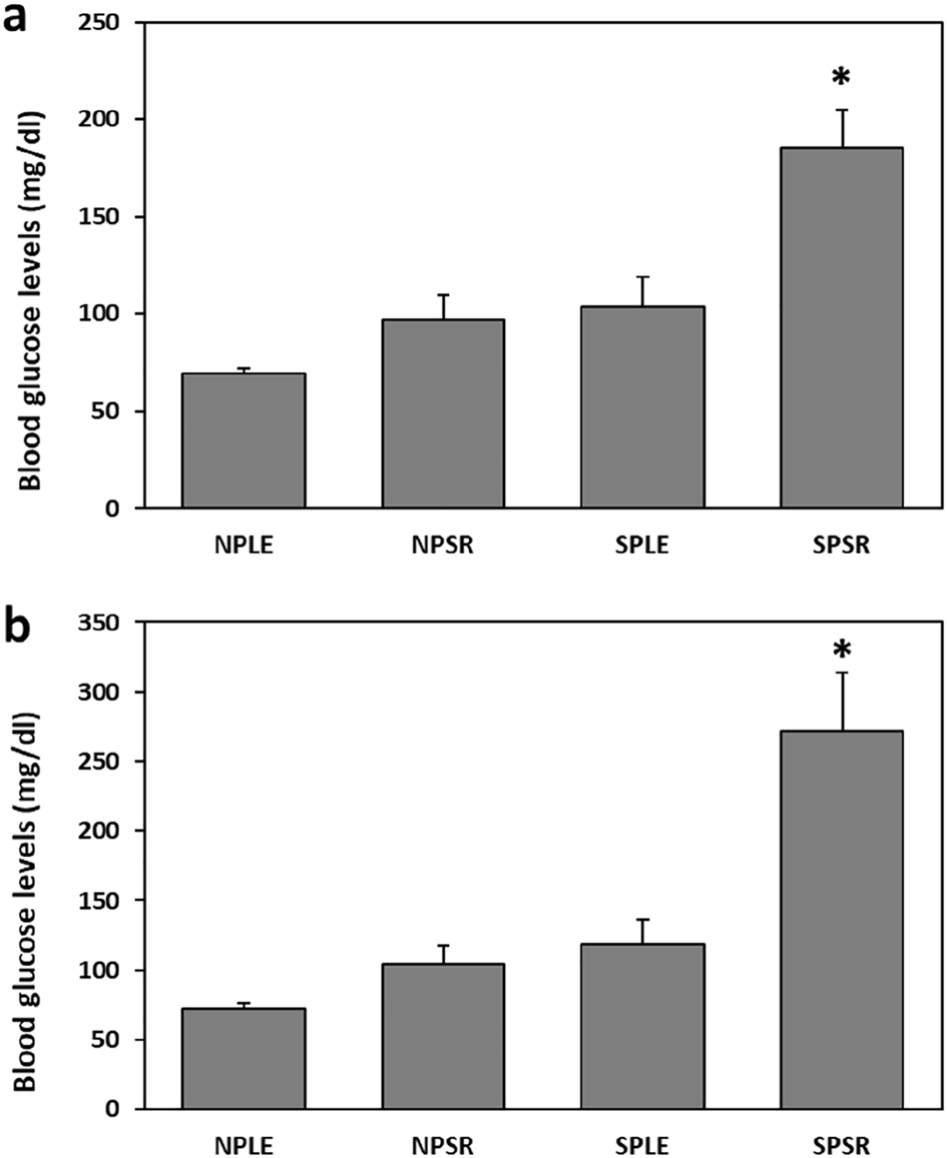
Blood glucose levels (mg/dl) at baseline (**a**) and 120 min after oral glucose administration (**b**) were significantly higher in sand rats acclimated to short photoperiod and fed standard rodent diet (SPSR) compared with all other groups (NPLE—neutral photoperiod with low energy diet, NPSR—neutral photoperiod with standard rodent diet, SPLE—short photoperiod with low energy diet). n = 11–12 per group, Mean ± SEM. *Signifies *p* < 0.001.

Animals kept under SP and fed SRD (SPSR) had significantly higher baseline blood glucose levels compared with all other groups, with a significant effect of both photoperiod [F(1, 43) = 22.124, *p* < 0.001] and diet [F(1, 43) = 13.353, *p* < 0.001], with no interaction [F(1, 43) = 2.749, *p* = 0.105](Fig. 4a). Post-hoc analysis: baseline blood glucose levels of SP animals with LED (SPLE) were significantly higher than those of NP animals with LED (NPLE) (*p* < 0.05), and significantly lower than those of SP animals with SRD (SPSR) (*p* < 0.001). Animals kept under SPSR had significantly higher baseline blood glucose levels than all other groups [NPLE—*p* < 0.001, NP with SRD (NPSR)—*p* < 0.001, SPLE—*p* < 0.01].

### Glucose tolerance

Animals kept under SPSR conditions had significantly higher blood glucose levels 120 min post glucose administration than all other groups. Both photoperiod and diet had a significant effect on glucose tolerance [Photoperiod: F(1,43) = 21.297, *p* < 0.001; Diet: F(1, 43) = 13.414, *p* < 0.001], with a significant interaction [F(1, 43) = 5.433, *p* < 0.05] (Fig. 4b). Post-hoc analysis: SPSR differs significantly from NPLE (*p* < 0.001), NPSR (*p* < 0.001), SPLE (*p* < 0.001).

### Number of cells in INL

**Figure 5:**
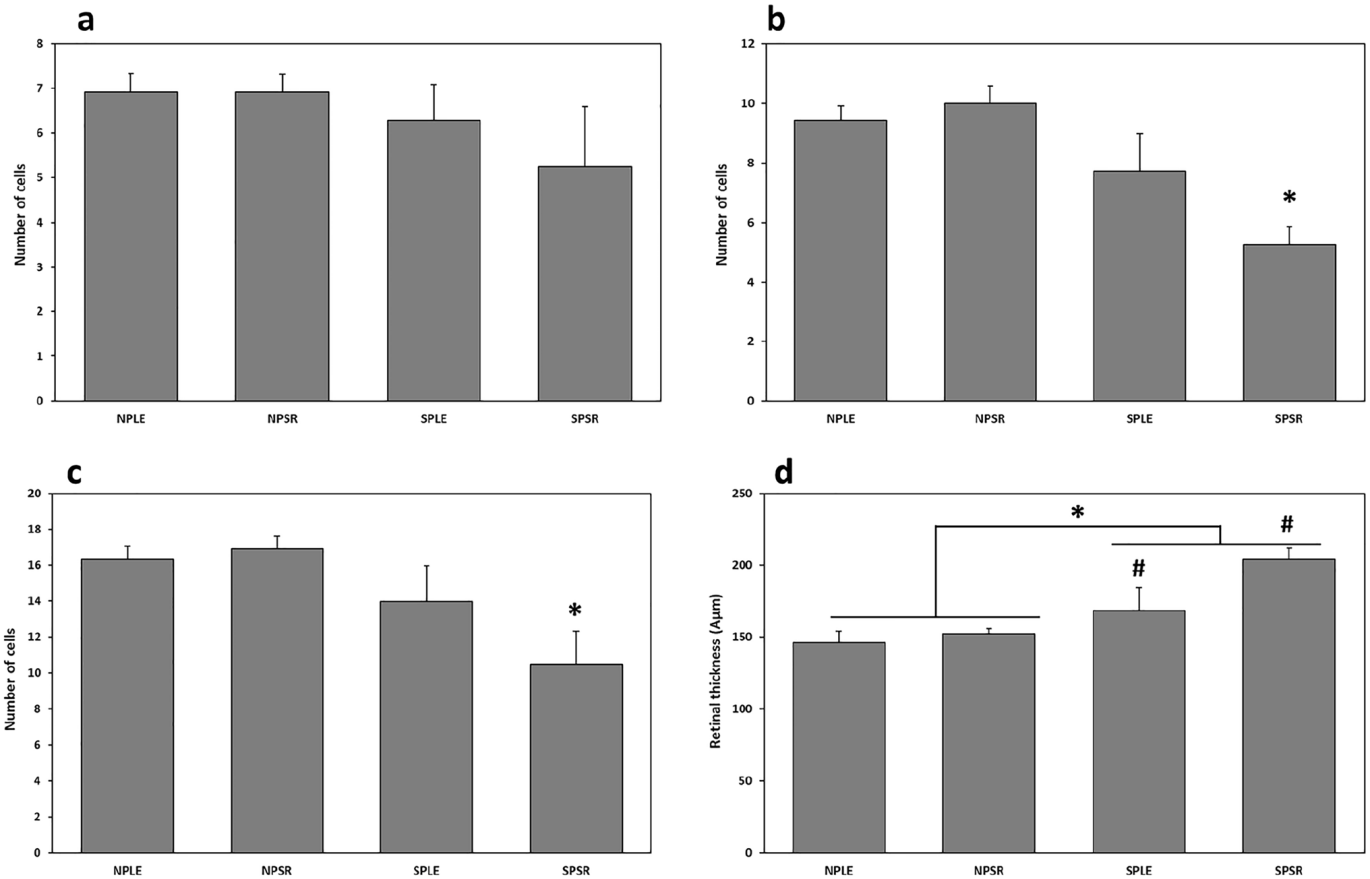
The number of cells in the INL (**a**) was similar in all groups, but the number of cells in the ONL (**b**) and the total number of cells in the retina (**c**) were significantly lower in sand rats acclimated to short photoperiod and fed standard rodent diet (SPSR) compared with other groups (NPLE—neutral photoperiod with low energy diet, NPSR—neutral photoperiod with standard rodent diet, SPLE—short photoperiod with low energy diet). Retinal thickness was significantly higher in short photoperiod acclimated animals of both SPLE and SPSR (**d**). n = 11–12 per group, Mean ± SEM. * signifies difference between NP and SP, *p* < 0.01. # signifies difference from all other groups, *p* < 0.05.

**Figure 6:**
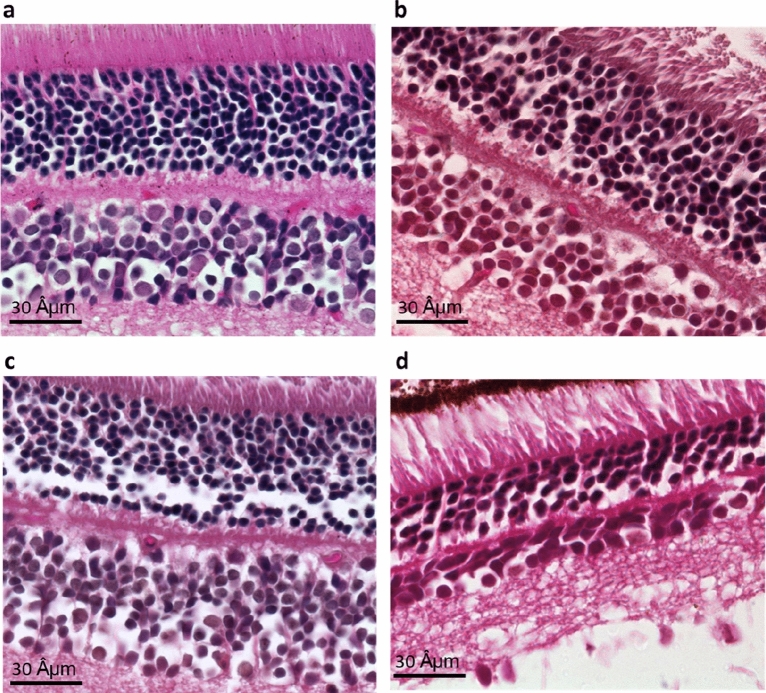
Micrograph of the INL and ONL of representative specimens from the NPLE group (**a**), NPSR group (**b**), SPLE group (**c**) and SPSR group (**d**). Stained with hematoxylin and eosin. Bar, 30 Âμm.

There was no effect of photoperiod or diet on the number of cells in the INL and no interaction [Photoperiod: F(1, 43) = 1.1919, *p* = 0.281; Diet: F(1, 43) = 0.941, *p* = 0.338; interaction: F(1, 43) = 0.941, *p* = 0.338](Fig. 5a, 6a–d).

### Number of cells in ONL

Photoperiod had a significant effect on the number of cells in the ONL [F(1, 43) = 15.185, *p* < 0.001], with no main effect of diet [F(1, 43) = 2.887, *p* = 0.097], but a significant interaction between diet and photoperiod [F(1, 43) = 6.163, *p* < 0.05]. Animals kept under SPSR conditions had significantly less cells in the ONL compared with all other groups (Fig. 5b, 6a–d). Post-hoc analysis: SPSR differs significantly from NPLE (*p* < 0.001), NPSR (*p* < 0.001), SPLE (*p* < 0.01).

### Total number of cells in the retina

Photoperiod had a significant effect on the total number of cells in the retina [F(1, 43) = 7.628, *p* < 0.01], with no effect of diet [F(1, 43) = 2.153, *p* = 0.15], and no significant interaction [F(1, 43) = 3.619, *p* = 0.064]. Animals kept under SPSR conditions had significantly less cells in the retina compared with all other groups (Fig. 5c, 6a–d). Post-hoc analysis: SPSR differs significantly from NPLE (*p* < 0.001), NPSR (*p* < 0.001), SPLE (*p* < 0.01).

### Retinal thickness

Photoperiod had a significant effect on retinal thickness, with SP animals having thicker retinas than NP animals [F(1, 43) = 44.963, *p* < 0.001], a borderline effect for diet [F(1, 43) = 3.94, *p* = 0.054] and no interaction [F(1, 43) = 1.177, *p* = 0.284] (Fig. 5d, 6a–d). Post-hoc analysis: SPSR differs significantly from NPLE (*p* < 0.001), NPSR (*p* < 0.001) and SPLE (*p* < 0.05). SPLE differs significantly from NPLE (*p* < 0.001) and NPSR (*p* < 0.01).

### Cataract

**Figure 7:**
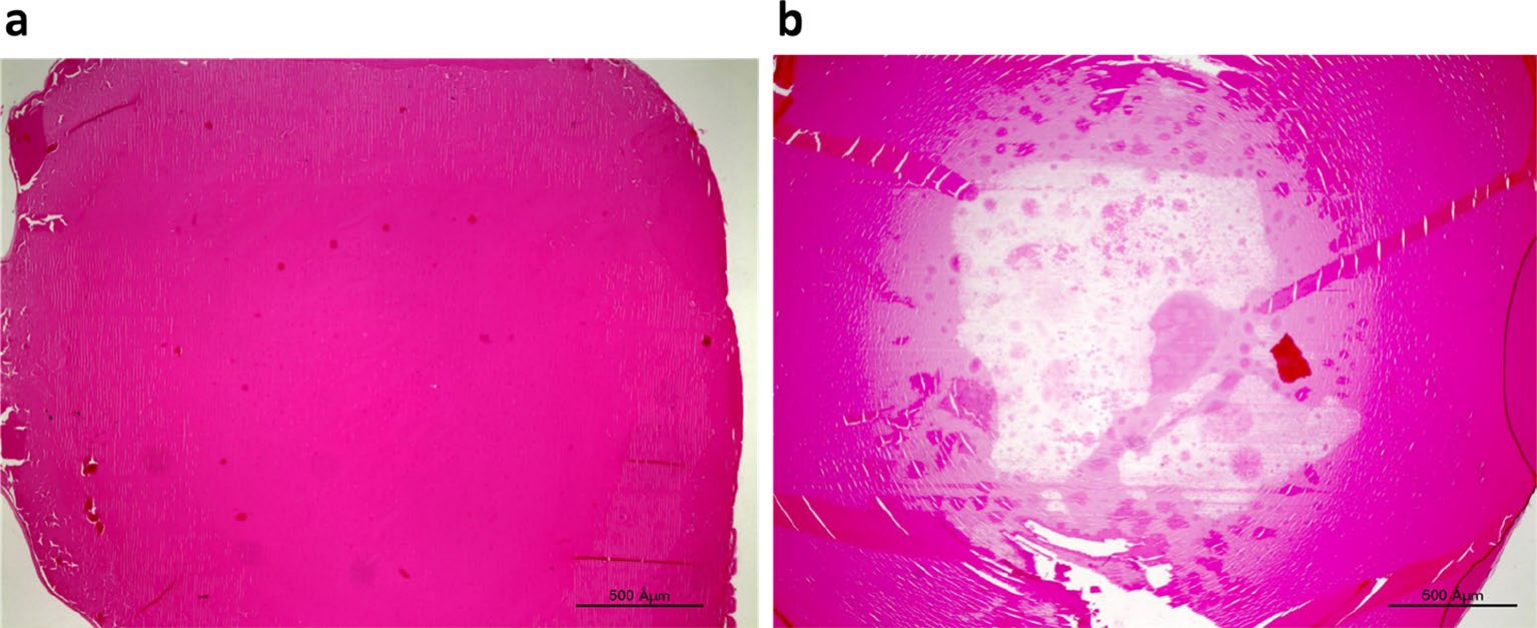
Micrograph of a clear lens of a specimen from the NPLE group (**a**) and a lens with mature cataract of a specimen from the SPSR group (**b**) stained with hematoxylin and eosin. Bar, 500 Âμm.

Both photoperiod and diet had a significant effect on the degree of cataract [Photoperiod: F(1, 43) = 24.090, *p* < 0.0001; Diet: F(1, 43) = 13.582, *p* < 0.001], with a significant interaction [F(1, 43) = 13.582, *p* < 0.001]. Animals kept under SPSR conditions had significantly more cataracts (NPLE—0% cataract, NPSR—0% cataract, SPLE—27% cataract, SPSR—67% cataract), and a higher degree of cataract compared with all other groups: only this group had a cataract degree of 2–3 (NPLE—cataract degree of 0 in all animals, NPSR—cataract degree of 0 in all animals, SPLE—3/11 animals showed cataract degree of 1, SPSR—1/12 animals showed cataract degree of 2 and 7/12 animals showed cataract degree of 3). Post-hoc analysis: SPSR differs significantly from NPLE (*p* < 0.001), NPSR (*p* < 0.001) and SPLE (*p* < 0.001) (Fig. 7a,b).

### Correlation between blood glucose levels in the OGT test and ocular structures

**Figure 8:**
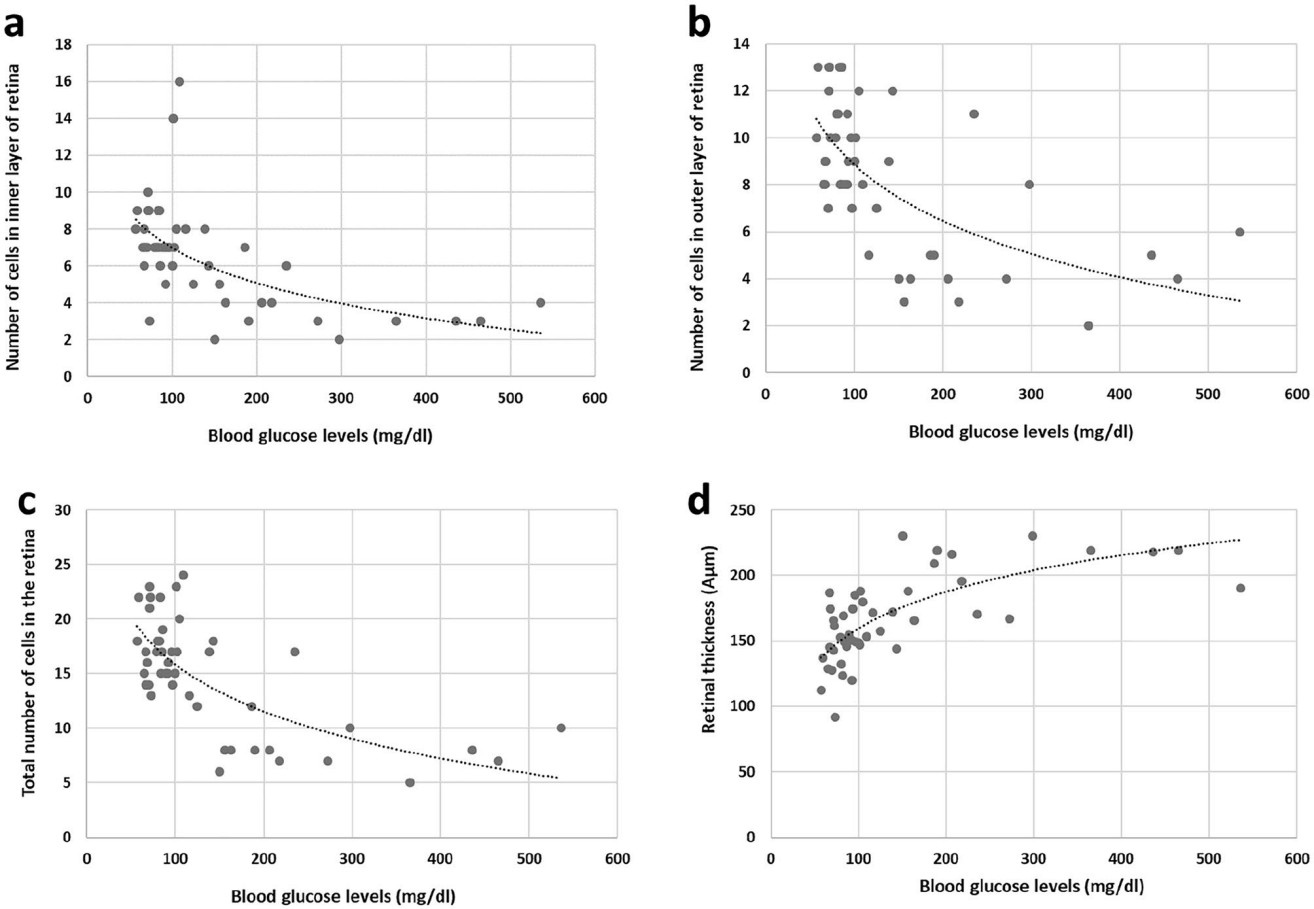
Correlations between blood glucose levels in the OGTT and the number of cells in the INL (**a**), ONL (**b**), the total number of cells in the retina (**c**) and retinal thickness (**d**). The dotted line represents a logarithmic model.

Higher blood glucose levels in the OGT test (OGTT) correlated significantly with a lower number of cells in the INL (R = − 0.551, *p* < 0.001, Fig. 8a), ONL (R = − 0.567, *p* < 0.001, Fig. 8b) and a lower total number of cells in the retina (R = − 0.642, *p* < 0.001, Fig. 8c), as well as with a thicker retina (R = 0.643, *p* < 0.001, Fig. 8d). A higher degree of cataract also correlated significantly with glucose levels in the OGTT (R = 0.836, *p* < 0.001).

## Discussion

The current study examined the effects of SP versus NP and LED versus SRD on glucose tolerance and ocular pathology in sand rats. Photoperiod had a significant effect on all parameters measured except for the number of cells in the INL. When combined with SRD, the effect was more pronounced. Standard laboratory conditions include constant ambient temperature, constant food availability, minimum mobility, no interspecific interactions, exposure to artificial light at night, and exposure to low light intensities during the day compared to natural daylight. These conditions are very similar to humans' modern living conditions^5,23^. The circadian disruption caused by these conditions can lead to a plethora of metabolic-related complications including T2DM, cataract and diabetic retinopathy ^5–7,18^. In sand rats, SP conditions accelerate the development of these complications.

Our results replicate previous findings and show that sand rats kept under SP conditions and fed SRD have significantly higher baseline blood glucose levels and lower glucose tolerance compared with animals kept under NP regardless of diet, and under SP with LED^7,18,24^. As in previous work, body weight does not differ significantly between the groups^7,18,24^. Additionally, we demonstrate that SP conditions led to significant retinal pathology, and the effect was larger when combined with SRD. SP conditions had a significant effect on the number of cells in the ONL, the total number of cells in the retina, retinal thickness and the degree of cataract. Moreover, SPSR animals conditions had significantly less cells in the ONL, a lower total number of cells in the retina, and a significantly thickened retina compared to SPLE animals. Furthermore, higher blood glucose levels correlated significantly with a lower number of cells in all layers of the retina and a higher thickness of the retina. These results could indicate the development of DR. The fact that the SPLE group, whose blood glucose levels and glucose tolerance did not differ from the NP groups, developed ocular pathology points to the direct effect of circadian disruption on its development. The development of more severe pathologies in the SPSR group, which showed significantly higher baseline blood glucose levels and lower glucose tolerance, and the correlation between higher blood glucose levels in the OGTT and the number of cells in all layers of the retina and a higher thickness of the retina, are consistent with previous findings showing neural abnormalities induced by T2DM in the laboratory rat and human retina^25^. These results also suggest an indirect effect of circadian disruption on retinal pathology, via the development of metabolic abnormalities. Altogether, our results suggest that circadian disruption has a combined direct and indirect (via the development of hyperglycemia and glucose intolerance) effect on ocular pathologies.

Although the effects of hyperglycemia on the development of DR have been studied extensively^26–29^, the effect of circadian rhythm disruptions on this pathology are less understood^6^. There is a marked decrease in all the clock genes (*Per2, Bmal1, Cry* and *Clock*) in the retina of rats with DR, as well as a dampened diurnal pattern of circulating angiogenic cells^30^. Furthermore, *Per2* dysfunction in mice recapitulates the phenotype of DR, with a decrease in retinal ERG function and overall retinal thickness^30^. In addition, conditional deletion of *Bmal1* in the endothelium leads to retinal microvascular and macrovascular injury and to retinopathy^31^. Furthermore, a variety of inflammatory markers exhibit altered circadian rhythm in DR. For example, endothelial cells isolated from donors with diabetes display an increased amplitude of IL-1 and ICAM-1^32^. The normal diurnal rhythm of autophagy proteins was lost with the induction of experimental DR in rats ^33^. Altogether, the results of these studies suggest that circadian rhythm disturbances may contribute to the disturbed retinal pathology observed in our study.

Evidence indicates that in DR, ischemic signals lead to excessive angiogenesis and neovascularization which are a major cause of blindness in T2DM patients^34^. This excess primes an increased risk of cardiovascular events—the growth of vasa vasorum inside the vascular wall is stimulated leading to bleeding, plaque instability and consequent rupture^35^. Even though we did observe some retinal pathology suggestive of DR in the SPSR sand rats, we found no clinical evidence of neovascularization in neither of the experimental groups. Future studies could examine which factor prevents neovascularization in the sand rats, despite the ischemic signal.

We further demonstrate that sand rats kept under SPSR conditions had significantly higher occurrence of cataract, and a higher degree of cataract compared with all other groups, and that the higher degree of cataract correlated with higher blood glucose levels in the OGTT.

These results are consistent with previous findings in sand rats, showing that animals develop hyperglycemia and cataract within 20 weeks of SP acclimation with SRD^7^. Hyperglycemia and diabetes are well-established risk factors for the development of cataract^36–38^. The exact mechanisms by which hyperglycemia contributes to cataract formation are not clear, but two possible mechanisms had been proposed: (a) Hyperglycemia may lead to high concentrations of glucose within the lens fibers leading to an accumulation of sorbitol in the lens. This may cause an imbalance in osmotic equilibrium and subsequent cataract formation^39^. Previous studies have shown significantly high level of sorbitol in the lens of humans which correlates with the degree of hyperglycemia^40^. (b) increased levels of glucose in the lens fibers result in accelerated nonenzymatic glycosylation and subsequent alteration of lens proteins, leading to cataract formation^41^. Either or both mechanisms could explain the association between hyperglycemia and the development and progression of cataracts^38^. These results demonstrate that SP conditions led to significant cataract development, and the effect was larger when combined with SRD.

Moreover, the statistical analysis shows that photoperiod length was a significant factor in most of the measures taken in this study including the number of cells in the ONL, total number of cells in the retina, retinal thickness and the degree of cataract, however, the diet was significant only for the degree of cataract. This suggests that the effect of circadian rhythm disturbances on the entire range of ocular pathologies may be higher than that of the diet.

The close similarity between the observed ocular pathologies in sand rats and those in humans with T2DM make the sand rat model valuable for studying the effects of modern lifestyle on circadian disruption-related diseases. Moreover, as a diurnal animal, their eyes structure and function are more similar to the human eye, and larger than traditional rodent models' eyes. We suggest that using diurnal model animals may be advantageous compared with nocturnal models in research that aims to understand the underlying mechanisms of the interactions between circadian rhythms and disease. Recognizing the link between modern lifestyle and circadian disruption, risk and etiology of T2DM and its severe, vision damaging, ocular complications, such as DR and cataract may have key implications for non-pharmacological prevention and therapeutic strategies to manage the contemporary and escalating epidemic of circadian rhythm-related diseases.

Although our results are robust, we should note some limitations: Intraocular pressure measurements using an immersive tonometer (Tonopen® XL, Medtronic Wolan, Jacksonville, Florida) were not reproducible, and therefore, were excluded. Furthermore, this study was performed using males only because of the significant changes in female physiology, possibly confounding the results. Future research should address additional pathophysiological aspects such as changes in the retinal blood plexuses and the choroid.

In this study, we examined the effect of photoperiod length and diet on the retina and lens of sand rats. Since some of the ocular pathologies we have shown, are evident in both SP groups, yet more severe in the SPSR group, it is presumable that they result from a combined effect of circadian disruption and diet. Future studies should address differences in ocular pressure, histopathology of the choroid and the flow of the retinal blood plexus.

## Data Availability

The datasets used and/or analysed during the current study are available from the corresponding author on reasonable request.

## References

[1] Michelle Chenault V, Ediger MN, Ansari RR (2002). In vivo assessment of diabetic lenses using dynamic light scattering. Diabetes Technol. Ther..

[2] Kiziltoprak H, Tekin K, Inanc M, Goker YS (2019). Cataract in diabetes mellitus. World J. Diabetes.

[3] Saïdi T (2011). The sand rat, *Psammomys obesus*, develops type 2 diabetic retinopathy similar to humans. Invest. Ophthalmol. Vis. Sci..

[4] Lutty GA (2013). Effects of diabetes on the eye. Invest. Ophthalmol. Vis. Sci..

[5] Zimmet P (2019). The Circadian Syndrome: Is the Metabolic Syndrome and much more!. J. Intern. Med..

[6] Bhatwadekar AD, Rameswara V (2020). Circadian rhythms in diabetic retinopathy: An overview of pathogenesis and investigational drugs. Expert Opin. Investig. Drugs.

[7] Bilu C (2019). Diurnality, type 2 diabetes, and depressive-like behavior. J. Biol. Rhythms.

[8] Verra DM, Sajdak BS, Merriman DK, Hicks D (2020). Diurnal rodents as pertinent animal models of human retinal physiology and pathology. Prog. Retin. Eye Res..

[9] Jeon C-J, Strettoi E, Masland RH (1998). The major cell populations of the mouse retina. J. Neurosci..

[10] Szél Á, Röhlich P (1992). Two cone types of rat retina detected by anti-visual pigment antibodies. Exp. Eye Res..

[11] Saidi T, Mbarek S, Ben Chaouacha-Chekir R, Hicks D (2011). Diurnal rodents as animal models of human central vision: Characterisation of the retina of the sand rat *Psammomys obsesus*. Graefes Arch. Clin. Exp. Ophthalmol..

[12] Kronfeld-Schor N (2001). Retinal structure and foraging microhabitat use of the golden spiny mouse (*Acomys russatus*). J. Mammal..

[13] Bilu C (2022). Effects of photoperiod and diet on BDNF daily rhythms in diurnal sand rats. Behav. Brain Res..

[14] Tan J (2019). High-energy diet and shorter light exposure drives markers of adipocyte dysfunction in visceral and subcutaneous adipose depots of *Psammomys obesus*. Int. J. Mol. Sci..

[15] Bilu C (2019). Red white and blue–bright light effects in a diurnal rodent model for seasonal affective disorder. Chronobiol. Int..

[16] Nankivell VA (2021). Circadian disruption by short light exposure and a high energy diet impairs glucose tolerance and increases cardiac fibrosis in *Psammomys obesus*. Sci. Rep..

[17] Madsen T, Ostercamp M (1982). notes on the biology of the fish eating snake (Lycodonomorphus bicolor) in lake Tanganyika. J. Herpatology.

[18] Bilu C, Kronfeld-Schor N, Zimmet P, Einat H (2022). Sex differences in the response to circadian disruption in diurnal sand rats. Chronobiol. Int..

[19] Ilan M, Yom-Tov Y (1990). Diel activity pattern of a diurnal desert rodent, *Psammomys obesus*. J. Mammal..

[20] Ilan M (1985). Aspects in the life-history of free-living sand rats *Psammomys obesus*. Isr. J. Zool..

[21] Bilu C (2022). Beneficial effects of voluntary wheel running on activity rhythms, metabolic state, and affect in a diurnal model of circadian disruption. Sci. Rep..

[22] Tzameret A (2017). Long-term safety of transplanting human bone marrow stromal cells into the extravascular spaces of the choroid of rabbits. Stem Cells Int..

[23] Stevenson TJ (2015). Disrupted seasonal biology impacts health, food security and ecosystems. Proc. R. Soc. B Biol. Sci..

[24] Bilu C (2019). Linking type 2 diabetes mellitus, cardiac hypertrophy and depression in a diurnal animal model. Sci. Rep..

[25] Lorenzi M, Gerhardinger C (2001). Early cellular and molecular changes induced by diabetes in the retina. Diabetologia.

[26] Klein R, Klein BEK, Moss SE, Cruickshanks KJ (1994). Relationship of hyperglycemia to the long-term incidence and progression of diabetic retinopathy. Arch. Intern. Med..

[27] Crawford TN, Alfaro DV, Kerrison JB, Jablon EP (2009). Diabetic retinopathy and angiogenesis. Curr. Diabetes Rev..

[28] Mbata O, El-Magd NFA, El-Remessy AB (2017). Obesity, metabolic syndrome and diabetic retinopathy: Beyond hyperglycemia. World J. Diabetes.

[29] Nyengaard JR, Ido Y, Kilo C, Williamson JR (2004). Interactions between hyperglycemia and hypoxia: Implications for diabetic retinopathy. Diabetes.

[30] Busik JV (2009). Diabetic retinopathy is associated with bone marrow neuropathy and a depressed peripheral clock. J. Exp. Med..

[31] Bhatwadekar AD (2017). Conditional deletion of Bmal1 accentuates microvascular and macrovascular injury. Am. J. Pathol..

[32] Wang Q (2014). Regulation of retinal inflammation by rhythmic expression of MiR-146a in diabetic retina. Invest. Ophthalmol. Vis. Sci..

[33] Qi X (2020). Diurnal rhythmicity of autophagy is impaired in the diabetic retina. Cells.

[34] Costa PZ, Soares R (2013). Neovascularization in diabetes and its complications. Unraveling the angiogenic paradox. Life Sci..

[35] Kota SK (2012). Aberrant angiogenesis: The gateway to diabetic complications. Indian J. Endocrinol. Metab..

[36] Donnelly CA (1995). Some blood plasma constituents correlate with human cataract. Br. J. Ophthalmol..

[37] Harding JJ, Harding RS, Egerton M (1989). Risk factors for cataract in Oxfordshire: Diabetes, peripheral neuropathy, myopia, glaucoma and diarrhoea. Acta Ophthalmol..

[38] Kanthan GL, Mitchell P, Burlutsky G, Wang JJ (2011). Fasting blood glucose levels and the long-term incidence and progression of cataract–the Blue Mountains Eye Study. Acta Ophthalmol..

[39] Jedziniak JA (1981). The sorbitol pathway in the human lens: Aldose reductase and polyol dehydrogenase. Invest. Ophthalmol. Vis. Sci..

[40] Varma SD, Schocket SS, Richards R (1979). Implications of aldose reductase in cataracts in human diabetes. Invest. Ophthalmol. Vis. Sci..

[41] Kasai K (1983). Increased glycosylation of proteins from cataractous lenses in diabetes. Diabetologia.

